# *UBE2D1* RNA Expression Was an Independent Unfavorable Prognostic Indicator in Lung Adenocarcinoma, but Not in Lung Squamous Cell Carcinoma

**DOI:** 10.1155/2018/4108919

**Published:** 2018-10-21

**Authors:** Liyan Hou, Yingbo Li, Ying Wang, Dongqiang Xu, Hailing Cui, Xiaoyan Xu, Ying Cong, Chengyong Yu

**Affiliations:** ^1^Department of Pharmacy, Weihai Central Hospital, Weihai, 264400 Shandong, China; ^2^Department of Blood Transfusion, Weihai Central Hospital, Weihai, 264400 Shandong, China; ^3^Clinical Laboratory, Weihai Central Hospital, Weihai, 264400 Shandong, China

## Abstract

In this study, we investigated the potential prognostic value of ubiquitin-conjugating enzyme E2D1 (*UBE2D1*) RNA expression in different histological subtypes of non-small-cell lung cancer (NSCLC). A retrospective study was performed by using molecular, clinicopathological, and survival data in the Cancer Genome Atlas (TCGA)—Lung Cancer. Results showed that both lung adenocarcinoma (LUAD) (*N* = 514) and lung squamous cell carcinoma (LUSC) (*N* = 502) tissues had significantly elevated *UBE2D1* RNA expression compared to the normal tissues (*p* < 0.001 and *p* = 0.036, respectively). *UBE2D1* RNA expression was significantly higher in LUAD than in LUSC tissues. Increased *UBE2D1* RNA expression was independently associated with shorter OS (HR: 1.359, 95% CI: 1.031–1.791, *p* = 0.029) and RFS (HR: 1.842, 95% CI: 1.353–2.508, *p* < 0.001) in LUAD patients, but not in LUSC patients. DNA amplification was common in LUAD patients (88/551, 16.0%) and was associated with significantly upregulated *UBE2D1* RNA expression. Based on these findings, we infer that *UBE2D1* RNA expression might only serve as an independent prognostic indicator of unfavorable OS and RFS in LUAD, but not in LUSC.

## 1. Introduction

Ubiquitination is a biological process, in which the targeting proteins were modified with ubiquitin for degradation [1]. This process is critical for cellular homeostasis, DNA repair, and proteasomal degradation [1]. Ubiquitination involves at least three classes of enzymes, including ubiquitin-activating enzymes (E1s), ubiquitin-conjugating enzymes (E2s), and ubiquitin-protein ligases (E3s) [1, 2]. E2s mediate ubiquitination by selective interactions with E1s and E3s and are responsible for the E3 selection and substrate modification, thus playing a critical role in ubiquitin transfer [3, 4]. They dictate the synthesis of a mono- or polyubiquitinated chain of a specific lysine linkage, which subsequently determines the fate of the substrate: proteasomal degradation or signaling [3, 4].

The ubiquitin-conjugating enzyme E2D family constitutes three members, including UBE2D1, UBE2D2, and UBE2D3, which all belong to the E2s. These family members share over 88% sequence identity and thus have similar enzymic activity [5, 6]. Previous studies found that UBE2D dysregulation is involved in some important pathways in carcinogenesis. They mediate the ubiquitination of the tumor-suppressor protein p53 [7–9]. Suppression of UBE2Ds can stabilize p53, leading to enhanced apoptosis and markedly inhibited proliferation of human lung cancer cells in a p53-dependent manner [10]. Among the three members, UBE2D1 can collaborate with cellular inhibitor of apoptosis protein 1 (c-IAP1) and mediate tumor necrosis factor *α*- (TNF*α*-) stimulated receptor-interacting protein 1 (RIP1) ubiquitination and NF-kappaB activation [11]. One recent study found that Smad ubiquitination regulatory factor 2 (SMURF2) and UBE2D1 form a critical E3 : E2 complex, which maintains Kirsten Ras (KRAS) protein stability [12]. Interruption of this complex by siRNA reduces the clonogenic survival *in vitro* and increases tumor latency *in vivo* in cancer cells including mutant KRAS-driven tumors [12]. These findings suggest that UBE2D1 might regulate some critical cancer-related signaling pathways.

In this study, by using the data from the Cancer Genome Atlas (TCGA)—Lung Cancer, we examined the expression profile of *UBE2D1* in the two major subtypes of non-small-cell lung cancer (NSCLC) lung adenocarcinoma (LUAD) and lung squamous cell carcinoma (LUSC) and its prognostic value in these subtypes.

## 2. Materials and Methods

### 2.1. Retrospective Analysis Using the Data from the Cancer Genome Atlas (TCGA)

This study is a retrospective study using the data from TCGA, with access provided by the UCSC Xena browser (https://xenabrowser.net/). In TCGA, molecular, clinicopathological, and over 10-year survival data of more than 500 LUAD or LUSC patients were recorded. Generally, in TCGA-LUAD, tumor tissues from 514 patients with primary tumors were collected for the RNA-seq study. 502 out of the 514 patients had intact OS data recorded. In TCGA-LUSC, tumor tissues from 501 patients with primary tumors were collected for the RNA-seq study. 494 out of the 501 patients had intact OS data recorded. Clinicopathological parameters of patients with primary LUAD, including age at diagnosis, gender, smoking history, clinical stage, nodal invasion, residual tumors, recurrence status, RFS in days, living status, and OS in days, were downloaded for survival-related analysis. Kaplan-Meier curves of OS and RFS were generated to examine the survival difference in patients with high/low *UBE2D1* RNA expression.

The genomic data, including *UBE2D1* DNA copy number alterations (CNAs), which were presented as gene-level thresholded GISTIC2-processed copy number data, were also downloaded to examine the association between *UBE2D1* RNA expression and the CNAs in LUAD patients.

### 2.2. Immunohistochemistry (IHC) Examination of *UBE2D1* Protein Expression

*UBE2D1* expression at the protein level in normal respiratory epithelial tissues and LUAD and LUSC tissues was characterized by using IHC staining data in the Human Protein Atlas (HPA) (https://www.proteinatlas.org/) [13, 14]. IHC scoring in the database was performed by combining staining intensity (negative, low, moderate, or strong) and fraction of stained cells (<25%, 25–75%, or >75%) [15]. Each combination of intensity and fractions is automatically converted into a protein expression level score as follows: not detected: negative (−), weak: <25%, low: weak combined with either 25–75% or 75%, moderate: <25%, medium: moderate combined with either 25–75% or 75%, strong: <25%, and high: medium/strong combined with either 25–75% or 75% [15].

### 2.3. Statistical Analysis

Statistical analysis was conducted by using GraphPad Prism 6.0 (GraphPad Inc., La Jolla, CA, USA) or SPSS 19.0 software package (SPSS Inc., Chicago, IL, USA). Welch's *t*-test was performed to examine the difference of *UBE2D1* RNA expression. Receiver operating characteristic (ROC) analysis for death and recurrence detection was performed to identify the Youden index for *UBE2D1* RNA expression (the cutoff to separate patients) in Kaplan-Meier curves of OS and RFS. The association between *UBE2D1* RNA expression and the clinicopathological parameters in LUAD patients was assessed by using the chi-squared test by two-sided Fisher's exact test. The log-rank test was conducted to examine the significance of the difference between the curves. Univariate and multivariate Cox regression models were used to evaluate the prognostic significance of *UBE2D1* RNA expression. *p* < 0.05 was considered statistically significant.

## 3. Results

### 3.1. *UBE2D1* RNA Expression Was Higher in LUAD and LUSC Tissues than in Normal Respiratory Tissues

**Figure 1:**
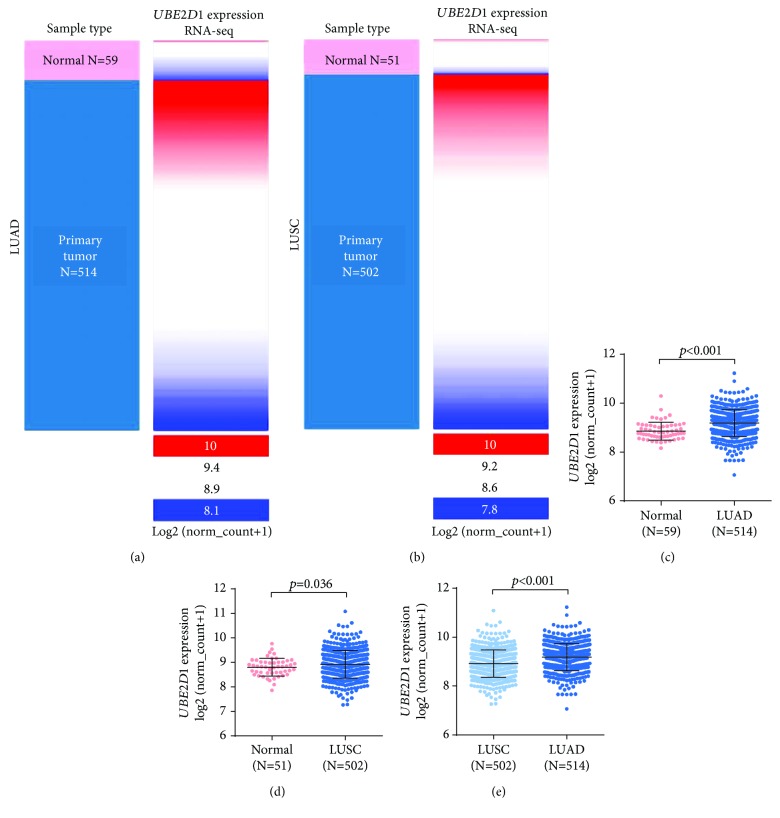
*UBE2D1* RNA expression was upregulated in both LUAD and LUSC tissues compared to normal lung tissues (a–d). Heatmap (a–b) and plot chart (b–c) of *UBE2D1* RNA expression in LUAD (*N* = 514) and LUSC (*N* = 502) tissues and their corresponding normal lung tissues. (e) Comparison of *UBE2D1* RNA expression between LUAD and LUSC tissues.

**Figure 2:**
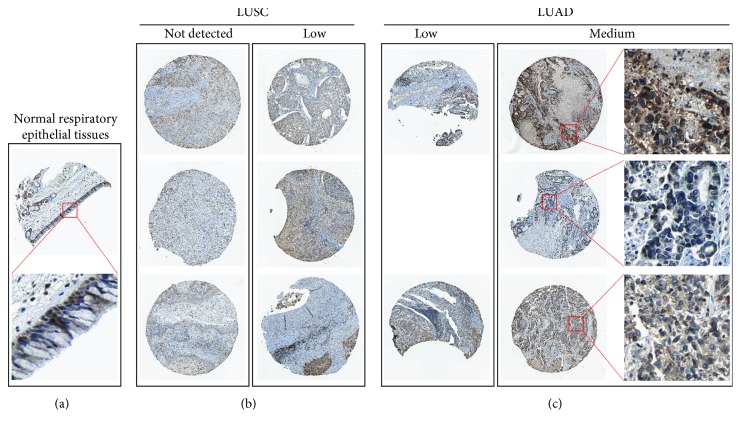
*UBE2D1* protein expression in normal respiratory epithelium and LUAD and LUSC tissues. *UBE2D1* IHC staining in normal respiratory epithelial tissues (a), in LUSC tissues (b), and in LUAD tissues (c).

By data mining in TCGA, we obtained the RNA-seq data of *UBE2D1* expression in NSCLC tissues and in normal lung tissues. The comparison showed that both LUAD (*N* = 514) and LUSC (*N* = 502) tissues had significantly elevated *UBE2D1* RNA expression compared to the normal tissues (*p* < 0.001 and *p* = 0.036, Figures 1(a)–1(d)). However, LUAD tissues had substantially higher expression of *UBE2D1* RNA expression compared to LUSC tissues (*p* < 0.001, Figure 1(e)). Using IHC staining data in the HPA, we also assessed *UBE2D1* expression at the protein level in cancer tissues and normal lung tissues. Results showed that normal respiratory tissues usually had low *UBE2D1* expression (Figure 2(a)). Among 6 cases of LUSC tissues examined, 3 cases had negative expression, while the rest 3 cases had low expression (Figure 2(b)). Among 5 cases of LUAD tissues examined, 2 cases had low expression and 3 cases had medium expression (Figure 2(c)). These findings suggest that *UBE2D1* might also be upregulated at the protein level in LUAD tissues.

### 3.2. Increased *UBE2D1* RNA Expression Was Associated with Poor Survival Outcomes in LUAD Patients, but Not in LUSC Patients

**Figure 3:**
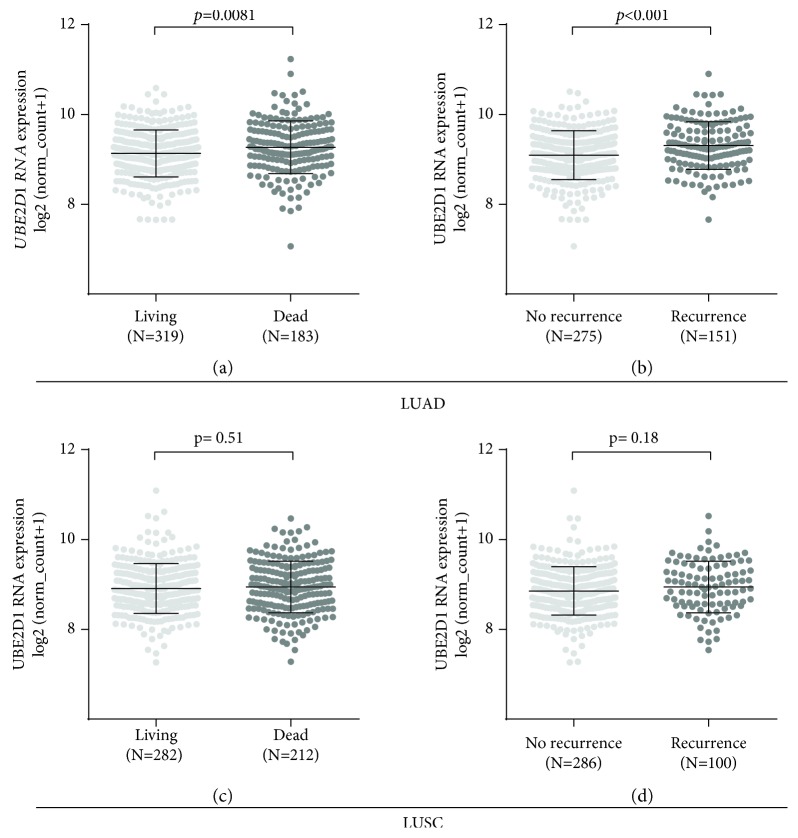
Comparison of *UBE2D1* RNA expression in LUAD/LUSC patients with different survival outcomes (a–d). Comparison of *UBE2D1* RNA expression in LUAD (a–b) and LUSC (c–d) patients according to their living status (a, c) and recurrence status (b, d).

**Figure 4:**
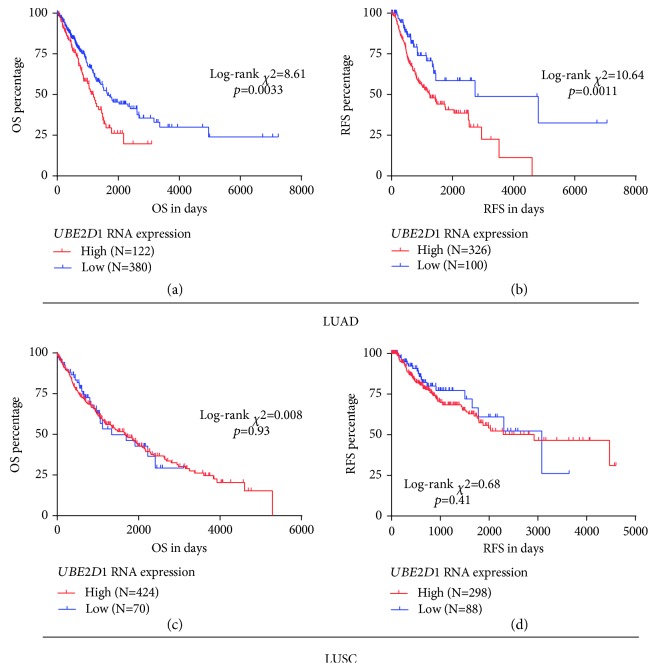
Kaplan-Meier curves of OS and RFS in LUAD and LUSC (a–d). Kaplan-Meier curves of OS (a, c) and RFS (b, d) in LUAD (a–b) and LUSC (c–d) patients. The patients were grouped according to the Youden Index identified in ROC analysis for death and recurrence detection.

Then, we assessed the association between *UBE2D1* RNA expression and survival outcomes in the major subtypes of NSCLC patients. The deceased LUAD cases (*N* = 183) had markedly higher *UBE2D1* RNA expression compared to the living cases (*N* = 319) (Figure 3(a)). Besides, the LUAD patients with recurrence (*N* = 151) also had significantly increased *UBE2D1* RNA expression compared to the patients without recurrence (*N* = 275) (Figure 3(b)). In comparison, we did not find any significant associations in LUSC (Figures 3(c) and 3(d)). Then, we generated Kaplan-Meier survival curves to examine the association between *UBE2D1* RNA expression and the survival outcomes. Results showed that in LUAD patients, the group with high *UBE2D1* RNA expression had inferior OS (*p* = 0.0033) and RFS (*p* = 0.0011) compared to the group with low expression (Figures 4(a) and 4(b)). In contrast, *UBE2D1* RNA expression was not associated with OS or RFS in LUSC patients (*p* = 0.93 and 0.41, respectively, Figures 4(c) and 4(d)).

### 3.3. *UBE2D1* RNA Expression Was Independently Associated with Shorter OS and RFS in LUAD Patients

The association between *UBE2D1* RNA expression and the clinicopathological parameters in *LUAD* patients is summarized in Table 1. The comparison showed that the group with high UBE2D1 RNA expression had a significantly higher ratio of patients in advanced stages (III/IV) (34/119 vs. 72/375, *p* = 0.04), nodal positive cases (50/120 vs. 117/371, *p* = 0.046), cases with residual tumors (8/91 vs. 8/261, *p* = 0.037), recurrence (48/101 vs. 103/325, *p* = 0.0043), and death (59/122 vs. 124/380, *p* = 0.0024) compared to the group with low UBE2D1 RNA expression.

In univariate analysis, we observed that advanced stages, positive nodal invasion with residual tumors, and increased *UBE2D1* RNA expression were associated with unfavorable OS and RFS in LUAD (Tables 2 and 3). The following multivariate analysis confirmed that increased *UBE2D1* RNA expression independently predicted poor OS (HR: 1.359, 95% CI: 1.031–1.791, *p* = 0.029) (Table 2) and RFS (HR: 1.842, 95% CI: 1.353–2.508, *p* < 0.001) (Table 3).

### 3.4. DNA CNAs Were Associated with Dysregulated *UBE2D1* RNA Expression in LUAD Patients

**Figure 5:**
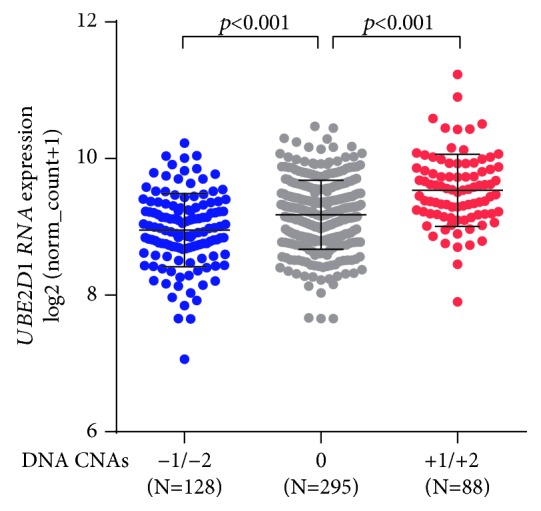
The association between *UBE2D1* CNAs and *UBE2D1* RNA expression. Comparison of *UBE2D1* RNA expression in different CNA groups. CNAs were defined as homozygous deletion (−2), heterozygous loss (−1), copy neutral (0), low-level copy gain (+1), and high-level amplification (+2).

Then, we explored the potential mechanism of *UBE2D1* dysregulation. Among 551 cases with both *UBE2D1* CNAs and RNA-seq data, 88 cases had amplification (+1/+2) CNA, 295 had copy neutral (0) CNA, and 128 had deletion (−1/−2) CNA (Figure 5). DNA deletion was associated with decreased *UBE2D1* RNA expression, while DNA amplification was associated with increased *UBE2D1* RNA expression, compared to the copy neutral group (Figure 5).

## 4. Discussion

In this study, our data mining results showed that although *UBE2D1* RNA was significantly upregulated in both LUAD and LUSC tissues compared with normal tissues, its expression was even higher in LUAD tissues than in LUSC tissues. Interestingly, we observed that *UBE2D1* RNA upregulation was associated with poor survival outcomes in LUAD patients, but not in LUSC patients. By performing univariate and multivariate analyses, we confirmed that increased *UBE2D1* RNA expression was independently associated with shorter OS (HR: 1.359, 95% CI: 1.031–1.791, *p* = 0.029) and RFS (HR: 1.842, 95% CI: 1.353–2.508, *p* < 0.001) in LUAD patients. Therefore, we infer that *UBE2D1* RNA expression might only serve as an independent prognostic indicator of unfavorable OS and RFS in LUAD, but not in LUSC.

Previous studies showed that UBE2Ds play a critical role in the ubiquitination and degradation of p53 [7, 8]. P53 is a master tumor-suppressive gene, and its degradation has a crucial role in human carcinogenesis, including NSCLC. P53 inactivation is closely associated with lung adenocarcinoma initiation, progression, and metastasis via multiple signaling pathways [16]. In Kras-driven LUAD, Notch1 initiates carcinogenesis by suppressing p53-mediated apoptosis through the regulation of p53 stability [17]. *P53* inactivation can lead to *DDX3* loss, which subsequently results in Slug-suppressed E-cadherin expression via decreased MDM2-mediated Slug degradation [18]. The NSCLC patients with *p53* inactivation also have poor survival outcomes [18]. P53 degradation directly lowers the p53-dependent transcription of the tumor suppressors *RAD51* and *p21* and the upregulation of the oncogene *SOX2* in LUAD [19]. A recent study showed that the UBE2Ds, together with RNF138, accumulate at damaged-DNA sites and promote DNA repair via promoting CtIP ubiquitylation and accrual [20]. In fact, the therapeutic effect of the current chemo- and radiotherapies mainly relies on inducing DNA damage of cancer cells. Therefore, upregulation of UBE2Ds in cancer cells may result in a weakened effect of the DNA-damaging anticancer therapy and rapid recovery after the damage. The key molecules in the DNA damage response (DDR) pathways have also been considered ideal targets for therapeutic intervention, including the E2s [21]. These mechanisms help explain why *UBE2D1* upregulation is associated with poor OS and RFS in LUAD patients.

Although we confirmed the potential prognostic value of *UBE2D1* RNA expression in LUAD, the mechanisms underlying its dysregulation have not been explored. In this study, we examined the association between *UBE2D1* RNA expression and the CNAs of its DNA, and the results showed that DNA amplification was common in LUAD patients (88/551, 16.0%) and was associated with significantly upregulated *UBE2D1* RNA expression, compared to the copy neutral patients. These findings suggest that DNA amplification might be an essential cause of upregulated *UBE2D1* in LUAD.

## 5. Conclusion

*UBE2D1* RNA expression might only serve as an independent prognostic indicator of unfavorable OS and RFS in LUAD, but not in LUSC. DNA amplification might be an essential cause of upregulated *UBE2D1* RNA expression in LUAD.

## Figures and Tables

**Table 1:** The associations between *UBE2D1* RNA expression and the clinicopathological parameters of patients with LUAD.

Parameters		*UBE2D1* RNA expression		*p* value
		High (*N* = 122)	Low (*N* = 380)	
Age (mean ± SD)		64.54 ± 10.99	65.58 ± 9.59	0.91

Gender	Female	65	206	0.92
	Male	57	174	

Smoking history	2/3/4/5	108	308	0.054
	1	11	61	
	No data	3	11	

Clinical stage	III/IV	34	72	**0.040**
	I/II	85	303	
	Discrepancy/no data	3	5	

Nodal invasion	N0	70	254	**0.046**
	N1/2/3	50	117	
	NX/no data	2	9	

Residual tumors	R0	83	253	**0.037**
	R1/R2	8	8	
	RX/no data	31	119	

Recurrence status	No	53	222	**0.0043**
	Yes	48	103	
	No data	21	55	

Living status	Living	63	256	**0.0024**
	Dead	59	124	

Smoking history: 1—lifelong nonsmoker, 2—current smoker, 3—current reformed smoker (for >15 yrs), 4—current reformed smoker (for ≤15 yrs), and 5—current reformed smoker (duration not specified); NX: regional lymph nodes cannot be assessed; RX: the presence of residual tumor cannot be assessed.

**Table 2:** Univariate and multivariate analyses of overall survival in patients with LUAD.

Parameters	Univariate analysis				Multivariate analysis			
	*p*	HR	95% CI (lower/upper)		*p*	HR	95% CI (lower/upper)	
Age (continuous)	0.330	1.008	0.992	1.023				
Gender (female vs. male)	0.670	0.939	0.702	1.256				
Smoking history (2/3/4/5 vs. 1)	0.662	0.912	0.604	1.377				
Clinical stage (III/IV vs. I/II)	**<0.001**	2.646	1.942	3.606	**0.005**	1.687	1.168	2.437
Nodal status (positive vs. negative)	**<0.001**	2.569	1.912	3.452	**<0.001**	1.906	1.346	2.700
Residual tumors (yes vs. no)	**<0.001**	3.937	2.204	7.033	**0.001**	2.650	1.463	4.801
*UBE2D1* RNA expression (continuous)	**0.007**	1.469	1.113	1.939	**0.029**	1.359	1.031	1.791

**Table 3:** Univariate and multivariate analyses of recurrence-free survival in patients with LUAD.

Parameters	Univariate analysis				Multivariate analysis			
	*p*	HR	95% CI (lower/upper)		*p*	HR	95% CI (lower/upper)	
Age (continuous)	0.323	1.008	0.992	1.025				
Gender (female vs. male)	0.574	1.097	0.794	1.516				
Smoking history (2/3/4/5 vs. 1)	0.435	1.208	0.752	1.939				
Clinical stage (III/IV vs. I/II)	**0.006**	1.711	1.168	2.506	**0.285**	1.288	0.810	2.048
Nodal status (positive vs. negative)	**0.003**	1.633	1.178	2.264	**0.183**	1.308	0.881	1.942
Residual tumors (yes vs. no)	**<0.001**	3.808	1.838	7.892	**0.008**	2.743	1.297	5.803
*UBE2D1* RNA expression (continuous)	**<0.001**	1.958	1.443	2.657	**<0.001**	1.842	1.353	2.508

## Data Availability

All TCGA data used in this study can be obtained via the UCSC Xena browser (https://xenabrowser.net/).
